# Prognostic factors in patients with acute mesenteric ischemia—novel tools for determining patient outcomes

**DOI:** 10.1007/s00464-022-09673-1

**Published:** 2022-10-10

**Authors:** Stefanie Sinz, Marcel A. Schneider, Simon Graber, Hatem Alkadhi, Andreas Rickenbacher, Matthias Turina

**Affiliations:** 1 Department of Surgery, University Hospital Zurich, Raemistrasse 100, CH-8091 Zurich, Switzerland; 2 Institute of Diagnostic and Interventional Radiology, University Hospital Zurich, Raemistrasse 100, CH-8091 Zurich, Switzerland; 3 Section of Colorectal Surgery, Department of Surgery, University Hospital of Zurich, Raemistrasse 100, CH-8091 Zurich, Switzerland

**Keywords:** Mesenteric ischemia, Pneumatosis intestinalis, Intestinal ischemia, Surgery

## Abstract

**Background:**

Acute mesenteric ischemia (AMI) is a devastating disease with poor prognosis. Due to the multitude of underlying factors, prediction of outcomes remains poor. We aimed to identify factors governing diagnosis and survival in AMI and develop novel prognostic tools.

**Methods:**

This monocentric retrospective study analyzed patients with suspected AMI undergoing imaging between January 2014 and December 2019. Subgroup analyses were performed for patients with confirmed AMI undergoing surgery. Nomograms were calculated based on multivariable logistic regression models.

**Results:**

Five hundred and thirty-nine patients underwent imaging for clinically suspected AMI, with 216 examinations showing radiological indication of AMI. Intestinal necrosis (IN) was confirmed in 125 undergoing surgery, 58 of which survived and 67 died (median 9 days after diagnosis, IQR 22). Increasing age, ASA score, pneumatosis intestinalis, and dilated bowel loops were significantly associated with presence of IN upon radiological suspicion. In contrast, decreased pH, elevated creatinine, radiological atherosclerosis, vascular occlusion (versus non-occlusive AMI), and colonic affection (compared to small bowel ischemia only) were associated with impaired survival in patients undergoing surgery. Based on the identified factors, we developed two nomograms to aid in prediction of IN upon radiological suspicion (C-Index = 0.726) and survival in patients undergoing surgery for IN (C-Index = 0.791).

**Conclusion:**

As AMI remains a condition with high mortality, we identified factors predicting occurrence of IN with suspected AMI and survival when undergoing surgery for IN. We provide two new tools, which combine these parameters and might prove helpful in treatment of patients with AMI.

Acute mesenteric ischemia (AMI) is an infrequent cause of acute abdominal complaints [1, 2] and due to the multitude of underlying factors, prediction of outcomes remains poor. As the duration of ischemia is associated with increasing mortality, rapid and correct diagnosis remains pivotal [3].

Based on the etiology, four types of AMI can be distinguished: embolic arterial occlusion (e.g., due to atrial fibrillation), thrombotic arterial occlusion (e.g., due to atherosclerosis), thrombotic venous occlusion (e.g., due to pancreatitis, liver fibrosis, or coagulation disorders), and non-occlusive MI (NOMI, e.g., due to excessive vasopressor requirements in ICU patients) [4]. However, in most cases the specific etiology remains unclear. Unfortunately, the value of clinical, biochemical, and radiological parameters is often limited due to a lack of diagnostic accuracy [5, 6]. Laboratory parameters such as D-dimers, c-reactive protein (CRP), leucocytes or lactate have limited specificity to aid in diagnosis [7–12]. Other parameters such as citrulline or intestinal fatty acid binding protein (I-FABP) with so far good discrimination in small patient series have not arrived in clinical practice yet [13].

For radiological detection, CT angiography with arterial and portal venous phase remains the gold standard [8, 14–16]. The classical textbook sign of intestinal ischemia is pneumatosis intestinalis (PI), which can however also be associated with other conditions [17, 18], so its prognostic relevance for AMI is under debate [19]. Further radiological signs potentially associated with AMI include bowel wall distension [20], reduced bowel wall enhancement, mesenteric edema or fat stranding, porto-mesenteric venous gas or free intraperitoneal air in case of perforation [21, 22]. However, most of these signs lack specificity or have insufficient inter-reader agreement [23].

Treating a patient with suspected AMI, surgeons are faced with two essential questions: (1) how likely does the individual patient have manifest AMI/IN and (2) what is the prognosis of patients with IN undergoing surgery?

The objective of the current study was therefore to identify factors predicting the presence of relevant AMI/IN in patients undergoing imaging for suspected AMI and factors predicting survival in patients undergoing surgery for AMI related IN. Based on those prognostic parameters, our goal was to provide treating physicians with decision guidance by development of new prognostic tools.

## Methods

### Study design and participants

The current study is a retrospective observational, monocentric analysis of patients undergoing radiological imaging for suspected AMI or surgery for confirmed AMI between January 1, 2014 and December 31, 2019 at the university hospital of Zurich (USZ), Switzerland. Patients were identified by reviewing all radiological studies during the study period for key words such as “mesenteric ischemia,” “bowel ischemia,” and “intestinal ischemia” in the suspected clinical or radiological diagnosis, while simultaneously the operation schedule was searched for patients who were operated for suspected MI during this period. This typically included patients presenting to emergency department with acute abdomen, sudden onset of abdominal pain, nausea and vomiting, sepsis or septic shock accompanied by common AMI risk factors in patients’ history or conspicuous laboratory markers were included in the study. Also, patients already hospitalized with increasing abdominal pain, especially after surgery (e.g., cardiac surgery) or intubated patients with increasing abdominal girth, increasing hemodynamic instability, and/or deteriorating laboratory parameters were reviewed. Patients with mechanical ileus or small bowel obstruction and consecutive necrotic bowel were not included in the analyses. Two members of the study team (S.S. & S.G.) reviewed medical, surgical, and imaging reports retrospectively. Data collection included baseline demographic data, comorbidities, vital parameters, radiological findings, laboratory values as well as operative variables, 30 day mortality and clinical outcomes of the index hospitalization. Laboratory values represent the most deviated value in the 24 h preceding the diagnosis. Part of the patients of this study were included also in a previous study on the predictive value of pneumatosis intestinalis in CT [19].

### Radiological studies

All patients included in the study underwent CT examinations, which were performed on a second or third generation dual-source CT scanner (SOMATOM Definition Flash or Force, Siemens Healthineers, Forchheim, Germany) using institutional multiphasic abdominal CT protocol settings including a non-enhanced, followed by an arterial and portal-venous phase CT scan (intravenous administration of 70–100 mL iodinated contrast media depending on the body weight with injection rate of 5 cc per second through an 18 gauge intravenous catheter). In seldom cases (e.g., acute renal failure or inaccurate application) native CT scans were performed. Standard image reconstructions were performed with a slice thickness of 2 mm (increment 1.5 mm) using a medium-soft tissue convolution kernel. Two radiologists (one with 4 years and one with 15 years of experience in abdominal imaging) independently evaluated all CT examinations. Images were reviewed for findings consistent with the most common signs of MI identified in previous literature, including bowel distension (defined as > 3 cm in small intestine, > 9 cm in caecum, and > 6 cm in the remaining colon), bowel wall thickening or hypo-enhancement, porto-mesenteric venous gas, free intraabdominal air and PI [21]. Ischemia was confirmed either surgically or endoscopically in patients with suspected AMI. Manifest AMI/IN was defined as either positive endoscopic findings such as necrotic mucosa or ischemic ulcers combined with radiological signs of AMI or positive intraoperative findings such as macroscopic necrotic, ischemic or livid bowel showing no signs of contraction or recovering blood flow intraoperatively. Patients with ischemic colitis and no clinical or radiological indication of IN were not included in the current study.

### Interventional and surgical parameters

Based on surgical reports, we recorded intraoperative findings such as the location and extent of ischemia, type of surgery, and surgeon’s level of education. Attempted preoperative recanalization was performed by interventional radiologists in a subgroup of patients with vascular occlusion and included mechanical/aspiration thrombectomy, thrombolysis, and/or percutaneous transluminal angioplasty and stenting.

### Ethics approval and written consent

The study adhered to and was conducted according to the principles of the Declaration of Helsinki and current good clinical practice guidelines. The protocol was approved by the responsible ethics committee of the canton of Zurich, Switzerland (BASEC-No. 2019–00208). Cases with a signed general consent were included in the study, while those with no permission to use their data were excluded. Collection of individual patients’ written consent was waived.

### Outcomes

The primary end point of the study was the assessment of survival rates of patients undergoing surgery for confirmed AMI/IN. Secondary endpoints were the rates of underlying AMI in patients with suspected ischemia, the identification of predictive factors for survival in patients undergoing surgery or for presence of AMI in patients undergoing imaging and the development of prediction tools based on the identified parameters. The aim of the study was the identification of above-mentioned risk factors to provide surgeons and radiologists with prognostic tools to aid in outcome stratification for patients with AMI.

### Statistical analysis

Statistical significance was defined as *p* < 0.05. Results are expressed as mean ± standard deviation (SD) or median ± interquartile range (IQR) as appropriate and were compared by Students *t*-test or Wilcoxon’s rank sum test as appropriate. Normal distribution of data was assessed with Shapiro–Wilk test. Correlation and collinearity between numerical variables was assessed with Pearson’s correlation coefficient. Categorical variables are presented as number (*n*) and percentage (%) and were compared with Fisher’s exact test. To identify factors influencing the presence of IN upon radiological suspicion or survival upon undergoing surgery for confirmed IN, multivariable logistic regression and respective odds ratios (OR) with 95% confidence intervals (CI) were computed. Goodness of fit of those models was assessed via the C-index according to Harrell [24] via internal validation of 200 bootstrapped samples as well as tenfold cross validation. The discriminative ability of the model was assessed with receiver-operating characteristic (ROC) curves, while agreement of observed frequencies and predicted probability of the models was assessed by calibration plots. Based on those models, nomograms [25] were developed to aid in future prediction of presence of ischemia or survival upon surgery respectively. *R* V4.0.2 and *R*-Studio V1.3.1093 (R Foundation for Statistical Computing, Vienna, Austria) were used for statistical analyses, calculations, and graphical representations [26].

## Results

### Characteristics of the overall radiological cohort

**Fig. 1:**
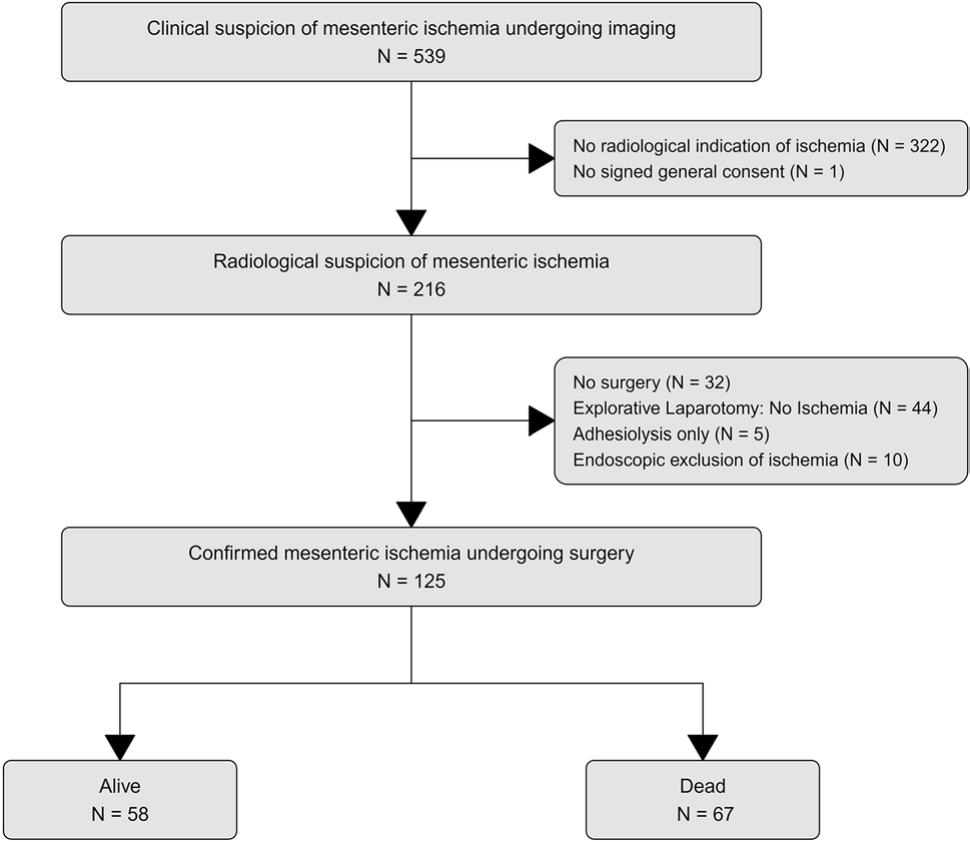
Flow diagram of patient selection

From January 1, 2014 to December 31, 2019, 539 patients (184/34.1% female and 355/65.9% male) with a median age of 65 years underwent radiological imaging for clinically suspected AMI. In 322 patients, there was no radiological indication of AMI, and one patient had no signed general consent. AMI was suspected in 216 patients. In 59 patients, ischemia was excluded upon surgical exploration (*n* = 44 explorative laparotomy with no ischemia, *n* = 5 adhesiolysis only) or endoscopically (*n* = 10) by macroscopically missing signs of bowel ischemia. In 20 patients no intervention was taken on diagnosis could not be definitively excluded or confirmed. In contrast, AMI with IN was definitively confirmed in 125 patients undergoing surgery. A flow chart of patient selection is depicted in Fig. 1.

### Predictive factors for presence of ischemia

In multivariable analyses, increasing age and ASA score as well as radiological pneumatosis intestinalis and dilated bowel loops were significantly associated with presence of IN upon radiological suspicion of AMI (Table 1). A personal history of peripheral vascular disease proofed significant in uni-, but not in multivariate analyses (OR 2.67, CI 1.25–6.23), while a history of heart disease and nicotine abuse as well as radiological signs of thickened bowel wall and atherosclerosis showed a borderline trend in univariate analyses. Further comorbidities such as atrial fibrillation (AF, OR 1.51, CI 0.85–2.68, *p* = 0.160) or diabetes mellitus type 2 (OR1.33, CI 0.68–2.70, *p* = 0.422) did not aid in distinguishing manifest AMI from other pathologies. All other factors explored in univariate analyses including gender, symptoms (nausea, vomiting, abdominal pain), vital parameters (pulse, systolic and diastolic blood pressure, body temperature, use of vasopressors), laboratory parameters (hemoglobin, leucocytes, CRP, creatinine, urea, pH, bicarbonate, lactate, aspartate aminotransferase (AST), alanine transaminase (ALT), albumin, phosphate, skeletal muscle creatine kinase (CK), myoglobin, lactate dehydrogenase (LDH)) as well as further radiological signs (free intraperitoneal air, mesenteric fluid, ascites, hyperdense, blurred or hypo-enhanced bowel wall, target sign, obstruction & ileus) showed no association with the presence of ischemia (all *p* > 0.1). Surprisingly, not even radiological stenosis or occlusion of a major abdominal vessel (celiac trunk, superior and inferior mesenteric artery, OR 0.92, CI 0.50–1.74, *p* = 0.802) or infarction of spleen, kidney, liver, or pancreas (1.61 (OR 1.61, CI 0.81–3.38, *p* = 0.189) could predict the occurrence of IN.

**Table 1:** Multivariate model of predictive factors for underlying ischemia in patients with radiologically suspected ischemia

Multivariate model: ischemia		No ischemia	Ischemia	OR (univariable)	OR (multivariable)
Age	Mean (SD)	62.6 (13.7)	66.6 (14.7)	1.02 (1.00–1.04, *p* = 0.049)	1.02 (1.00–1.04, *p* = 0.047)
ASA-score	1–3	20 (37.0)	34 (63.0)		
	4–5	28 (21.9)	100 (78.1)	2.10 (1.04–4.20, *p* = 0.036)	2.76 (1.26–6.19, *p* = 0.012)
Heart disease	No	35 (43.8)	45 (56.2)		
	Yes	44 (32.4)	92 (67.6)	1.63 (0.92–2.88, *p* = 0.094)	
Peripheral arterial disease	No	70 (40.7)	102 (59.3)		
	Yes	9 (20.5)	35 (79.5)	2.67 (1.25–6.23, *p* = 0.015)	1.88 (0.71–5.46, *p* = 0.221)
Nicotine abuse	No	36 (43.9)	46 (56.1)		
	Suspended	16 (28.6)	40 (71.4)	1.96 (0.96–4.11, *p* = 0.070)	
	Persistent	26 (35.6)	47 (64.4)	1.41 (0.74–2.72, *p* = 0.294)	
Radiological: pneumatosis intestinalis	No	48 (44.0)	61 (56.0)		
	Yes	31 (29.5)	74 (70.5)	1.88 (1.07–3.33, *p* = 0.029)	2.91 (1.31–6.80, *p* = 0.011)
Radiological: atherosclerosis	No	25 (46.3)	29 (53.7)		
	Yes	54 (34.0)	105 (66.0)	1.68 (0.89–3.14, *p* = 0.107)	
Radiological: bowel distension	No	41 (48.2)	44 (51.8)		
	Yes	38 (29.5)	91 (70.5)	2.23 (1.27–3.96, *p* = 0.006)	3.62 (1.67–8.20, *p* = 0.001)
Radiological: thickening bowel wall	No	36 (31.6)	78 (68.4)		
	Yes	43 (43.0)	57 (57.0)	0.61 (0.35–1.07, *p* = 0.085)	

### A nomogram for prediction of ischemia

**Fig. 2:**
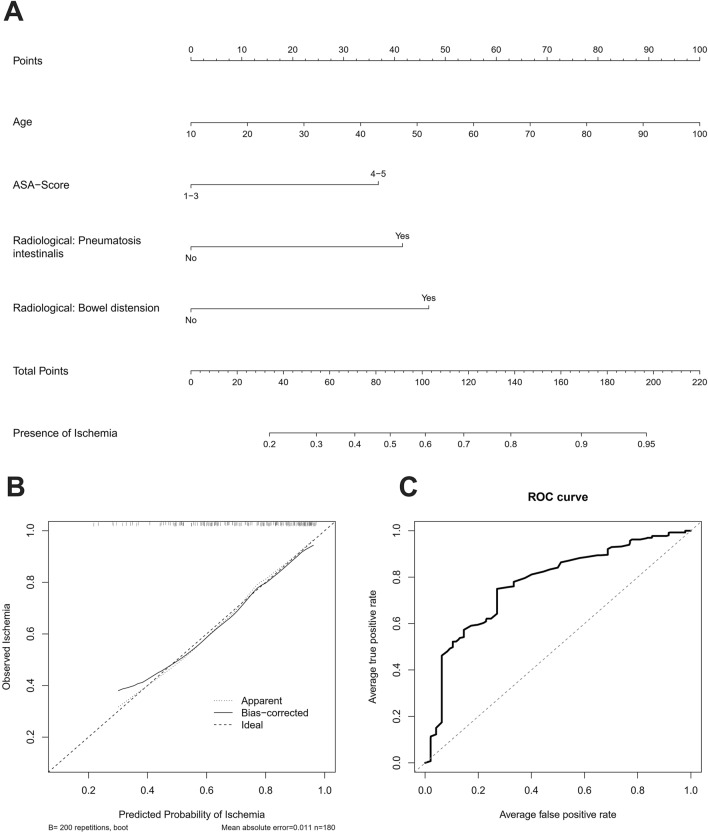
**A** Nomogram for prediction of ischemia based on clinical and radiological variables. **B** Calibration plot. **C** ROC Curve

Based on the remaining multivariate significant factors for relevant AMI, we developed a nomogram to predict the presence of ischemia (Fig. 2A). The nomogram and the underlying multivariate model showed an adequate calibration (Fig. 2B) and good discrimination (Fig. 2C) with a C-Index of 0.726 upon internal validation with bootstrapping and tenfold cross validation and provided an adequate stratification to predict ischemia.

### Characteristics of the surgical cohort and predictive factors for survival

Of the patients 137 patients with confirmed IN, 125 underwent surgery of which 67 (53.6%) died in the postoperative course (median 9 days after diagnosis, IQR 22) and 58 (46.4%) survived. Patients surviving were of equal age and had similar frequencies of heart disease or AF, but less often underlying diabetes and a reduced ASA score (Table 2). There was no significant difference with respect to symptoms (nausea, vomiting, abdominal pain, all *p* > 0.1), other comorbidities (nicotine abuse, peripheral arterial disease), and vital parameters (pulse, systolic and diastolic blood pressure, body temperature, use of vasopressors, all *p* > 0.1). Patients succumbing to the disease postoperatively showed decreased hemoglobin at presentation (*p* = 0.004), increased creatinine (*p* = 0.003), decreased pH (*p* ≤ 0.001) and bicarbonate (*p* ≤ 0.001) as well as increased lactate (*p* = 0.001). Inflammatory parameters such as leucocytes (*p* = 0.537) or CRP (*p* = 0.922) as well as any other biochemical markers were not different between survivors and non-survivors. Vascular occlusion was more frequent in non-survivors (arterial stenosis 19.4%, arterial occlusion 16.4%, venous occlusion 1.5%, no occlusion 62.7) compared to survivors (arterial stenosis 3.4%, arterial occlusion 8.6%, no occlusion 87%, *p* = 0.008).

**Table 2:** Baseline characteristics of patients undergoing surgery for confirmed MI

	Survivors (*N* = 58)	Non-Survivors (*N* = 67)	*P*-value	Total (*N* = 125)
Gender				
Female	19 (32.8%)	25 (37.3%)	0.708	44 (35.2%)
Male	39 (67.2%)	42 (62.7%)		81 (64.8%)
Age (years)				
Median [Q1, Q3]	68.0 [53.3, 78.0]	70.0 [63.0, 77.0]	0.079	70.0 [58.0, 78.0]
ASA-Score				
1	0 (0.0%)	0 (0.0%)	< 0.001	0 (0.0%)
2	7 (12.1%)	0 (0.0%)		7 (5.6%)
3	18 (31.0%)	7 (10.4%)		25 (20.0%)
4	29 (50.0%)	51 (76.1%)		80 (64.0%)
5	4 (6.9%)	9 (13.4%)		13 (10.4%)
Diabetes mellitus				
No	49 (84.5%)	45 (67.2%)	0.038	94 (75.2%)
Type2	9 (15.5%)	21 (31.3%)		30 (24.0%)
Missing	0 (0%)	1.00 (1.5%)		1.00 (0.8%)
Heart disease				
No	25 (43.1%)	18 (26.9%)	0.062	43 (34.4%)
Yes	33 (56.9%)	49 (73.1%)		82 (65.6%)
Atrial fibrillation				
No	35 (60.3%)	34 (50.7%)	0.367	69 (55.2%)
Yes	23 (39.7%)	33 (49.3%)		56 (44.8%)
Peripheral arterial disease				
No	44 (75.9%)	49 (73.1%)	0.838	93 (74.4%)
Yes	14 (24.1%)	18 (26.9%)		32 (25.6%)
Nicotine abuse				
No	19 (32.8%)	24 (35.8%)	0.826	43 (34.4%)
Stopped	15 (25.9%)	19 (28.4%)		34 (27.2%)
Persistent	22 (37.9%)	22 (32.8%)		44 (35.2%)
Missing	2.00 (3.4%)	2.00 (3.0%)		4.00 (3.2%)
Nausea				
No	37 (63.8%)	41 (61.2%)	0.894	78 (62.4%)
Yes	13 (22.4%)	17 (25.4%)		30 (24.0%)
Intubated/Gastric tube	6 (10.3%)	9 (13.4%)		15 (12.0%)
Missing	2.00 (3.4%)	0 (0%)		2.00 (1.6%)
Vomiting				
No	32 (55.2%)	37 (55.2%)	1	69 (55.2%)
Yes	15 (25.9%)	19 (28.4%)		34 (27.2%)
Intubated	5 (8.6%)	6 (9.0%)		11 (8.8%)
Reflux gastric tube	4 (6.9%)	5 (7.5%)		9 (7.2%)
Missing	2.00 (3.4%)	0 (0%)		2.00 (1.6%)
Abdominal pain				
No	16 (27.6%)	25 (37.3%)	0.181	41 (32.8%)
Yes	38 (65.5%)	34 (50.7%)		72 (57.6%)
Sedated	3 (5.2%)	8 (11.9%)		11 (8.8%)
Missing	1.00 (1.7%)	0 (0%)		1.00 (0.8%)
Vascular occlusion				
No	44 (75.9%)	39 (58.2%)	0.008	83 (66.4%)
Arterial stenosis	2 (3.4%)	13 (19.4%)		15 (12.0%)
Arterial occlusion	5 (8.6%)	11 (16.4%)		16 (12.8%)
Venous occlusion	0 (0.0%)	1 (1.5%)		1 (0.8%)
Missing	7.00 (12.1%)	3.00 (4.5%)		10.0 (8.0%)
Type of surgery				
Small bowel resection	25 (43.1%)	19 (28.4%)	0.051	41 (32.8%)
Colectomy	21 (36.2%)	33 (49.3%)		54 (43.2%)
Small bowel resection & colectomy	12 (20.7%)	11 (16.4%)		23 (18.4%)
Open/close—too much ischemia	0 (0.0%)	4 (6.0%)		4 (3.2%)
Second look surgery				
No	28 (48.3%)	29 (43.3%)	0.299	57 (45.6%)
Planned	24 (41.4%)	24 (35.8%)		48 (38.4%)
On demand	6 (10.3%)	14 (20.9%)		20 (16.0%)
Undergone interventional radiological revascularization				
No	46 (79.3%)	48 (71.6%)	0.296	94 (75.2%)
Yes	12 (20.7%)	16 (23.9%)		28 (22.4%)
Unsuccessful	0 (0.0%)	3 (4.5%)		3 (2.4%)

### Characteristics of interventional and surgical procedures

Median duration between imaging and surgery was similar for surviving (337 min) and deceased (300 min, *p* = 0.45) patients. Interventional radiological revascularization was used with equal frequency in both groups, with three unsuccessful interventions among diseased patients (*p* = 0.296). Deceased patients needed colon resection more often and in four patients, ischemia was so extensive that the operation was terminated after laparotomy. Other surgical characteristics had no influence upon survival. Open abdomen treatment was performed equally frequent among survivors (37.9%) and non-survivors (52.2.%, *p* = 0.15). Second look surgery was performed in > 50% of both groups, with no differences in planned or on-demand procedures (*p* = 0.299). A minimally invasive approach was attempted more often in survivors (25.9% vs. 10.4%, *p* = 0.024), with conversion to open surgery following detection of ischemia in all but one patient (1.7%). Survival was not influenced by the presence of a senior consultant at the operating table of the index operation (*p* = 0.448), nor were outcomes impaired during nights or weekends compared to regular weekdays (*p* = 0.701).

### Predictive factors for survival upon undergoing surgery

The above identified parameters were subsequently included in a multivariable model. Due to strong collinearity between pH and bicarbonate (*R* = 0.8, *p* ≤ 0.001), pH and lactate (*R* = − 0.73, *p* ≤ 0.001) as well as bicarbonate and lactate (*R* = − 0.65, *p* ≤ 0.001) as markers of metabolic acidosis, we included only pH as the overall surrogate marker of disturbed acid–base metabolism and shock. Furthermore, ASA score was not included in the multivariable model as a higher score per se predicts a higher mortality. The final model showed increasing creatinine levels, decreasing pH as well as three radiological signs, namely atherosclerosis, vascular occlusion, and colonic ischemic involvement, as predictors for impaired survival (Table 3). Diabetes mellitus type 2, decreased hemoglobin and radiological infarction of liver, pancreas, spleen, or kidney proved significant in univariate analyses, but not in the multivariate model, while age showed a borderline trend toward impaired survival (*p* = 0.076).

**Table 3:** Multivariate model of predictive factors for survival in patients undergoing surgery for confirmed MI

Multivariate model: survival		Survivors	Non-survivors	OR (univariable)	OR (multivariable)
Age	Mean (SD)	64.5 (16.9)	69.2 (12.2)	1.02 (1.00–1.05, *p* = 0.076)	1.02 (0.99–1.07, *p* = 0.225)
ASA-Score	1–3	25 (78.1)	7 (21.9)		
	4–5	33 (35.5)	60 (64.5)	6.49 (2.65–17.76, *p* < 0.001)	4.58 (1.42–16.29, *p* = 0.014)
DM	No	49 (51.6)	46 (48.4)		
	Type2	9 (30.0)	21 (70.0)	2.49 (1.06–6.23, *p* = 0.042)	1.45 (0.47–4.73, *p* = 0.524)
Hemoglobin	Mean (SD)	106.5 (28.7)	93.5 (18.8)	0.98 (0.96–0.99, *p* = 0.005)	0.99 (0.97–1.01, *p* = 0.370)
Creatinine	Mean (SD)	116.8 (64.6)	157.7 (84.4)	1.01 (1.00–1.01, *p* = 0.005)	1.01 (1.00–1.01, *p* = 0.029)
pH	Mean (SD)	7.4 (0.1)	7.3 (0.1)	0.00 (0.00–0.06, p = 0.001)	0.00 (0.00–0.11, *p* = 0.005)
Radiological: Atherosclerosis	No	23 (76.7)	7 (23.3)		
	Yes	35 (36.8)	60 (63.2)	5.63 (2.29–15.44, *p* < 0.001)	6.62 (2.25–22.47, *p* = 0.001)
Radiological: Infarction of Liver, Spleen, Kidney, or Pancreas	No	50 (53.2)	44 (46.8)		
	Yes	8 (25.8)	23 (74.2)	3.27 (1.37–8.48, *p* = 0.010)	2.70 (0.84–9.74, *p* = 0.107)
Radiological: Vascular occlusion	No	51 (54.8)	42 (45.2)		
	Yes	7 (21.9)	25 (78.1)	4.34 (1.78–11.78, *p* = 0.002)	3.48 (1.24–10.89, *p* = 0.023)
Location of ischemia	Small Intestine only	25 (61.0)	16 (39.0)		
	Colonic Involvement	33 (39.3)	51 (60.7)	2.41 (1.13–5.27, *p* = 0.024)	3.02 (1.21–7.98, *p* = 0.021)

### A nomogram for prediction of survival

**Fig. 3:**
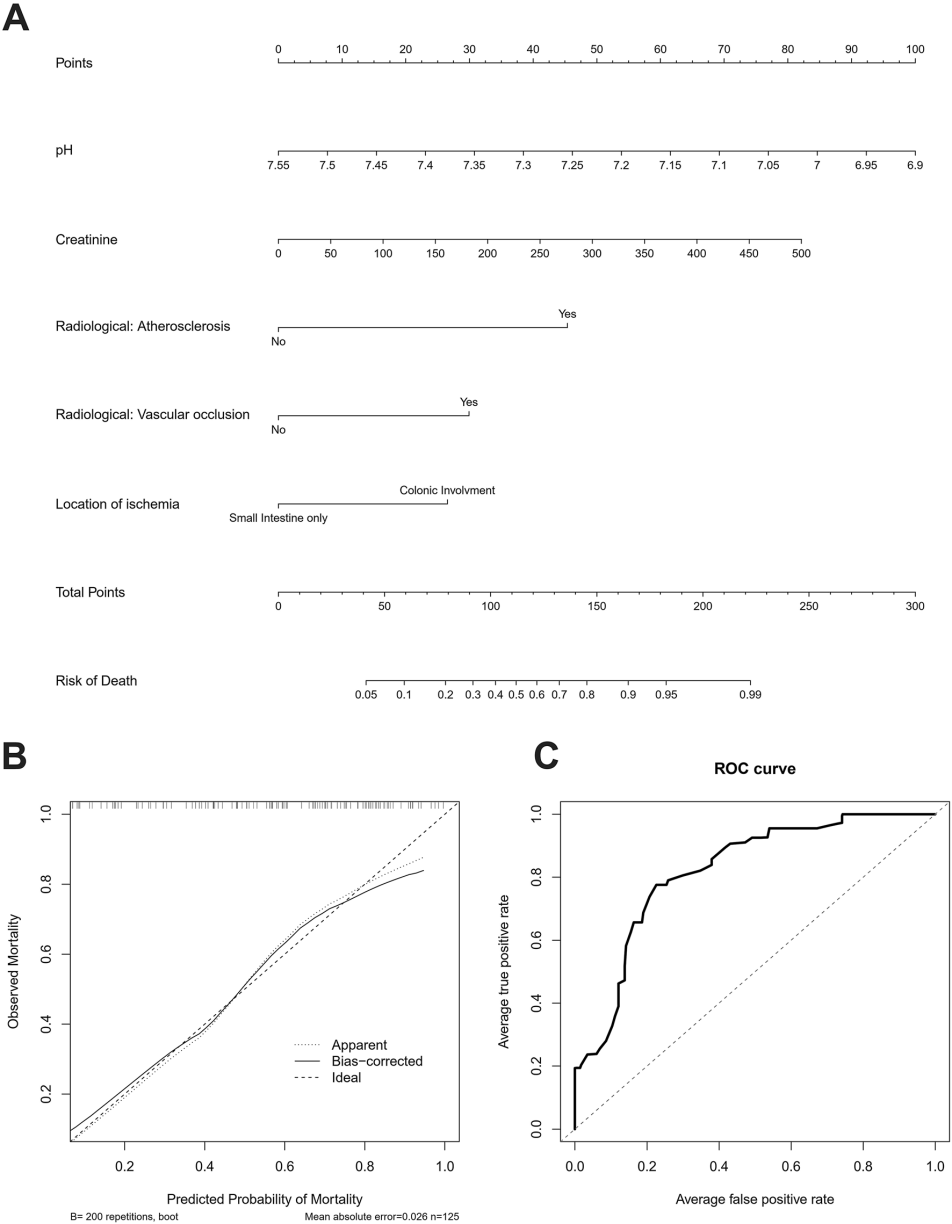
**A** Nomogram for prediction of survival in patients undergoing surgery for MI based on laboratory and radiological variables. **B** Calibration plot. **C** ROC Curve

Based on the identified factors for survival in the multivariable model, we developed a nomogram to determine the likelihood of survival in patients undergoing surgery for IN (Fig. 3A). The nomogram and the underlying model showed an adequate calibration (Fig. 3B) and good discrimination (Fig. 3C) with a C-Index of 0.791 upon internal validation with bootstrapping and tenfold cross validation and provided adequate survival stratification.

## Discussion

AMI remains a devastating condition with a broad clinical presentation and high lethality. Our nomograms based on an extensive retrospective patient dataset combining clinical signs and symptoms, biochemical laboratory parameters and radiological findings, allow identification of patients at risk for IN or death upon undergoing surgery.

The first nomogram enables the identification of patients with a high probability of underlying ischemia and need for acute surgical exploration. The factors relevant for prediction of IN are increasing age and ASA score as well as radiological signs of PI and bowel distension. The estimation of survival at time of index surgery is enabled by use of our second nomogram, which includes decreased pH, increased creatinine, radiological atherosclerosis, vascular occlusion (versus non-occlusive MI) and involvement of the colon.

Multiple previous studies have examined the diagnostic and predictive value of biochemical or radiological parameters to determine outcomes in AMI [8]. None of the currently available routine laboratory markers showed an association with the presence of IN upon radiological suspicion of AMI in our study. Lactate is probably the most frequently discussed prognostic marker in routine clinical practice. While there is an association between radiological PI, elevated lactate levels and manifest AMI [18], lactate remains a highly unspecific maker of tissue hypoperfusion rather than a specific marker of IN [27]. In our analyses, lactate did not prove helpful to determine whether IN is present in case of suspected AMI. However, high lactate levels, which correlated strongly with a decreased pH and depleted bicarbonate as a sign of shock, predicted, not surprisingly, decreased survival. Specific markers for intestinal ischemic damage, as used in clinical practice for cardiac ischemia, are so far lacking and discussed candidates such as citrulline, I-FABP, and d-lactate lack sufficient discriminative ability or mandate further testing [11, 28]. AST has been discussed as sensitive indicator of tissue necrosis and has been described as an independent predictor of death in patients with AMI [29, 30]. Our study could not confirm an association of elevated liver enzymes and the presence of IN, in line with the findings of limited sensitivity and specificity of liver enzymes in diagnosis of AMI by other authors [9]. Renal insufficiency, e.g., increased creatinine, has previously been associated with impaired outcomes after AMI [31]. As deterioration of renal function can be caused by various factors like dehydration, renal hypoperfusion [32] or shock with subsequent multiple organ failure, creatinine level at admission were not useful for the diagnosis of AMI. Similar to lactate and pH, increasing creatinine levels also predispose to lethality in patients undergoing surgery for IN. In contrast, inflammatory markers such as CRP or leukocyte count did not prove helpful in diagnosing patients with AMI, in accordance with a recent systematic review which showed high variation in sensitivity (57.1% to 90%) as well as specificity (36.5% to 100%) for leucocytes [9].

In addition to unreliable biomarkers, most radiological findings for AMI and manifest IN lack sensitivity and specificity and further hamper the diagnosis. While AMI is the most frequent etiology for PI, especially in combination with reduced bowel wall enhancement or porto-mesenteric venous gas [33, 34], other pathologies can confer this radiological sign and it does not by itself mandate an operative approach [35–37]. This fact is reflected in our findings, as 29% of patients with radiological PI had no detectable IN/mucosal ischemia. The most reliable and reproducible finding seems to be reduced enhancement of the bowel wall [23], while in our and other analyses distension of the bowel predicted IN. Colonic involvement in AMI seems to be associated with impaired outcomes [38], which was also identified in our cohort. Our analyses also bear certain surprising findings, as the occlusion of major abdominal vessels or infarction of spleen, kidney, liver, or pancreas did not predict the occurrence of AMI. Cases of occluded vessels with no IN might be owed to chronic mesenteric ischemia with sufficient collateral vascularization, while infarctions in parenchymatous abdominal organs can be related to various other pathologies. Furthermore, atrial fibrillation, the best-known risk factor for embolic AMI, was not significantly associated with occurrence of manifest AMI or survival.

Based on previously identified predictive factors, several prognostic tools to aid in management of AMI have been developed. Wayne et al. reported an algorithm using abdominal pain, small bowel distension, lactic acid levels, and presence of vascular disease to distinguish between AMI and benign causes of PI [18]. Another multicenter study developed a model using three variables (lactate > 2 mmol/l, presence of peritonitis, and vasopressor use) for estimating presence of IN in PI [39]. Umpathi et al. developed a nomogram to predict IN upon laparotomy for AMI including lactate levels, tenderness, tachycardia, and distended bowel loops [40]. Zhuang et al. published a score based on blood urea nitrogen, leukocytes, and d-dimers to predict IN [41], while colleagues from France identified organ failure, lactate > 2 mmol/l, and bowel distension to aid in diagnosis of irreversible IN [6]. Several of the identified parameters correspond to our findings, e.g., bowel distension, which was also identified in three of the above mentioned reports. However, none of these scores have made it into clinical practice so far, probably due to the intricate usage.

Over the last decade, the treatment of AMI has shifted from open surgical approaches with embolectomy, bypass, and resection of avital intestine to a multidisciplinary approach comprising hybrid and endovascular interventional procedures for restoration of blood flow with adequate results [42, 43] followed by laparoscopy or laparotomy viability assessment and resection of necrotic bowel as well as damage control surgery if necessary [1, 15, 44–46]. Surgical bypass in the acute setting is performed seldomly nowadays, while the value of newer techniques such as retrograde open mesenteric stenting (ROMS) remains to be evaluated [47, 48]. Interestingly, in our analyses, we did not observe a survival difference of patients with confirmed IN undergoing revascularization versus no intervention, the reason for this remains elusive.

The general surgical approach in AMI to assess viability and resection of necrotic tissue has historically been performed by laparotomy. Minimally invasive surgical approaches have not yet gained widespread acceptance in treatment of confirmed MI. While general guidelines for abdominal emergencies suggest laparoscopy if no diagnosis can be found by conventional diagnostics to avoid negative laparotomy [49, 50], specific current guidelines do not support the routine use of laparoscopy in AMI [51]. Laparoscopy might have limited ability to assess bowel viability and might be challenging, especially in unexperienced hands, and the risk of missing segmental areas of nonviable bowel must be considered in therapeutic decision-making. To increase the sensitivity in laparoscopic surgery for bowel ischemia, new methods are increasingly available, like fluorescein use for illustrating bowel perfusion [52]. In stable patients, we support the initial use of laparoscopy to confirm or exclude the diagnosis. Upon diagnosis of IN, however, therapeutic laparoscopy with bowel resection remains challenging, therefore, an open approach is usually inevitable. This is also mirrored in our data, where conversion upon laparotomy was performed in all but one patient upon detection of ischemia. With our nomograms, we aim to facilitate the decision between open and minimally invasive surgery, so patients with low predicted risk of IN, bowel viability may first be assessed by laparoscopy, while in patients with high predicted risk laparotomy is indicated.

The current study is limited by its retrospective, single-center nature and all the inherent potential biases associated with such a design. Furthermore, citrulline, D-Dimers, and I-FABP, which among biochemical parameters have shown the best discrimination for diagnosis of AMI/IN, are not routinely measured at our institution, and could therefore not be assessed in our analyses. Lastly, our models and nomograms lack external validation of their predictive abilities in separate individual cohorts, which should be performed in future studies.

In summary, our report provides a comprehensive analysis of patients undergoing imaging for suspected AMI and outcomes of patients with confirmed IN undergoing surgery. We identified factors predicting occurrence of IN, namely increasing age and ASA score, PI, and bowel distension. Parameters affecting survival when undergoing surgery for AMI include colonic involvement, vascular occlusion, atherosclerosis, increasing creatinine and decreasing pH.

Lastly, we provide two valuable new tools, which combine those parameters and might prove helpful to predict occurrence of IN and survival outcomes after surgery in patients with AMI and can easily be incorporated into daily practice.
